# Supplementary material for: Insight predicts subsequent memory via cortical representational change and hippocampal activity
Source: Nat Commun. 2025 May 9;16:4341. doi: 10.1038/s41467-025-59355-4 (PMC12064812; doi:10.1038/s41467-025-59355-4)
Supplement: Supplementary file 2 — Reporting Summary [file 41467_2025_59355_MOESM2_ESM.pdf]

nature portfolio

Corresponding author(s): Dr. Maxi Becker

Last updated by author(s): 26/March/2025

Reporting Summary

Nature Portfolio wishes to improve the reproducibility of the work that we publish. This form provides structure for consistency and transparency in reporting. For further information on Nature Portfolio policies, see our Editorial Policies and the Editorial Policy Checklist.

Statistics

For all statistical analyses, confirm that the following items are present in the figure legend, table legend, main text, or Methods section.

n/a	Confirmed	
☐	☒	The exact sample size (*n*) for each experimental group/condition, given as a discrete number and unit of measurement
☐	☒	A statement on whether measurements were taken from distinct samples or whether the same sample was measured repeatedly
☐	☒	The statistical test(s) used AND whether they are one- or two-sided<br>*Only common tests should be described solely by name; describe more complex techniques in the Methods section.*
☐	☒	A description of all covariates tested
☐	☒	A description of any assumptions or corrections, such as tests of normality and adjustment for multiple comparisons
☐	☒	A full description of the statistical parameters including central tendency (e.g. means) or other basic estimates (e.g. regression coefficient) AND variation (e.g. standard deviation) or associated estimates of uncertainty (e.g. confidence intervals)
☐	☒	For null hypothesis testing, the test statistic (e.g. F, t, r) with confidence intervals, effect sizes, degrees of freedom and P value noted<br>*Give P values as exact values whenever suitable.*
☒	☐	For Bayesian analysis, information on the choice of priors and Markov chain Monte Carlo settings
☐	☒	For hierarchical and complex designs, identification of the appropriate level for tests and full reporting of outcomes
☐	☒	Estimates of effect sizes (e.g. Cohen's *d*, Pearson's *r*), indicating how they were calculated

Our web collection on statistics for biologists contains articles on many of the points above.

Software and code

Policy information about availability of computer code

Data collection	Stimulus data set (Mooney images): https://github.com/MaxiBecker/Insight_Memory_Effect (v.20)
Data analysis	The analysis code is publicly available and can be accessed in the Zenodo database https://doi.org/10.5281/zenodo.14743957

For manuscripts utilizing custom algorithms or software that are central to the research but not yet described in published literature, software must be made available to editors and reviewers. We strongly encourage code deposition in a community repository (e.g. GitHub). See the Nature Portfolio guidelines for submitting code & software for further information.

Data

Policy information about availability of data

All manuscripts must include a data availability statement. This statement should provide the following information, where applicable:

- Accession codes, unique identifiers, or web links for publicly available datasets
- A description of any restrictions on data availability
- For clinical datasets or third party data, please ensure that the statement adheres to our policy

The raw behavioural data including the aggregated univariate and multivariate fMRI data, as well as the analysis code have been made publicly available and can be accessed at https://github.com/MaxiBecker/Insight_Memory_Effect (v.20). The raw fMRI data are not publicly available due to data privacy regulations. However, de-identified pre-processed fMRI data can be made available upon request from the corresponding author, subject to institutional and ethical approvals.

Research involving human participants, their data, or biological material

Policy information about studies with human participants or human data. See also policy information about sex, gender (identity/presentation), and sexual orientation and race, ethnicity and racism.

Reporting on sex and gender	The full sample consisted of 38 participants [age (in years): range = 20 – 34, 23 females: M= 26.6; 15 males: M= 25.7] recruited via an online student platform in Berlin. The final sample consisted of N=31 [age (in year): range = 19 – 33, 20 females: M= 25.13; 11 males: M= 26.27]. (see data exclusion). Female/male refers to the participant's sex which they indicated in a short online survey (to test for MRI compatibility, see below) prior to study invitation. In all analyses, we aggregated across both sexes as we did not have a specific research question regarding neither sex differences.
Reporting on race, ethnicity, or other socially relevant groupings	Inclusion criteria were German as mother language, normal or corrected to normal vision, between 18 - 35 years, no prior neurological or psychiatric diseases and MRI compatibility, which the participant indicated themselves in an online survey before being invited to the study (note, MRI compatibility was additionally assessed orally by a physician before starting the MRI sessions). All participants who met those conditions were invited to the study irregardless of race, ethnicity or any other socially relevant grouping. We did not have a specific research question regarding differences between race, ethnicity etc. in our study. For this reason, we did not ask the participants to provide us with this information.
Population characteristics	see above
Recruitment	The full sample consisted of 38 participants recruited via an online student platform in Berlin.
Ethics oversight	The local ethics committee of the Humboldt University Berlin approved the study (proposal number 2020-48).

Note that full information on the approval of the study protocol must also be provided in the manuscript.

Field-specific reporting

Please select the one below that is the best fit for your research. If you are not sure, read the appropriate sections before making your selection.

☐ Life sciences ☒ Behavioural & social sciences ☐ Ecological, evolutionary & environmental sciences

For a reference copy of the document with all sections, see nature.com/documents/nr-reporting-summary-flat.pdf

Life sciences study design

All studies must disclose on these points even when the disclosure is negative.

Sample size	*Describe how sample size was determined, detailing any statistical methods used to predetermine sample size OR if no sample-size calculation was performed, describe how sample sizes were chosen and provide a rationale for why these sample sizes are sufficient.*
Data exclusions	*Describe any data exclusions. If no data were excluded from the analyses, state so OR if data were excluded, describe the exclusions and the rationale behind them, indicating whether exclusion criteria were pre-established.*
Replication	*Describe the measures taken to verify the reproducibility of the experimental findings. If all attempts at replication were successful, confirm this OR if there are any findings that were not replicated or cannot be reproduced, note this and describe why.*
Randomization	*Describe how samples/organisms/participants were allocated into experimental groups. If allocation was not random, describe how covariates were controlled OR if this is not relevant to your study, explain why.*
Blinding	*Describe whether the investigators were blinded to group allocation during data collection and/or analysis. If blinding was not possible, describe why OR explain why blinding was not relevant to your study.*

Behavioural & social sciences study design

All studies must disclose on these points even when the disclosure is negative.

Study description	This is an experimental mixed method study with a within-subject design. We studied various quantitative fMRI measures (univariate activity and multivariate analyses: RSA, functional connectivity and graph measures) as a function of insight. This insight measure, however, is a subjective qualitative measure and cannot be directly experimentally controlled. We used a median split measure to divide the insight measure into high and low insight trials (the absolute value may differ between participants).
Research sample	The full sample consisted of 38 participants [age (in years): range = 20–34, 23 females: M = 26.6; 15 males: M = 25.7] recruited via an online student platform in Berlin. Inclusion criteria were German as mother language, normal or corrected to normal vision, between 18 – 35 years, no prior neurological or psychiatric diseases and MRI compatibility. Consent was obtained for all participants prior to study begin and all participants received monetary compensation according to their time on task. The study protocol was in accordance with the protocol of Helsinki. After excluding 7 participants from data analysis this resulted in a final sample of N=31 [age

(in year): range = 19 – 33, 20 females: M= 25.13; 11 males: M= 26.27]. Note, while the platform was also accessible to non-students, the sample remains somewhat selective regarding age and educational background. To ensure a higher degree of similarity among participants in terms of reaction times and specifically brain-related measures, we implemented an age restriction. This was intended to reduce inter-individual variability and enhance sensitivity for detecting effects. Self-selection bias: Participants who enjoy problem-solving may have self-selected into the study, potentially inflating the overall solution likelihood and affecting generalizability. However, the within-subject design, where participants generated their own control condition (LO-I), makes it unlikely that this bias would systematically influence the comparison between HI-I and LO-I, and thus the main research question.

Sampling strategy

A convenience sampling procedure was applied as we predominantly recruited among university students. No specific sample size calculation was performed.
No statistical method was used to predetermine sample size due to the absence of prior multivariate fMRI results on insight. Based on previous research (Kizilirmak et al., 2016; Ludmer et al., 2011) 20 participants would suffice for behavioral and univariate fMRI effects. To account for the lower power of multivariate fMRI, we aimed for a final sample size of approximately 30.
(Kizilirmak et al., 2016; PsychResearch: https://doi.org/10.1007/s00426-015-0697-2; Ludmer et al., 2011, Neuron: DOI 10.1016/j.neuron.2011.02.013).

Data collection

Participants had two MRI sessions [on a Siemens Magnetom Prisma 3T scanner (Erlangen, Germany) using a standard 64-channel head coil] on two consecutive days and in each session they completed two runs with 30 trials each. For the MRI experiments, the same two individuals were always present alongside the participant: the researcher (MRI operator) responsible for the study and a student assistant. The presented images per run and session were counterbalanced between participants to avoid order effects. Before entering the MRI scanner, participants were instructed and received three test trials to assure they understood the task. The paradigm was presented on a black screen (resolution 1280*960 pixel) using Matlab (2016a) and Psychtoolbox-3 (v3.0.17; Kleiner et al., 2007). The black/white Mooney images had a size of 800x800 pixels. During each trial a Mooney image was presented for 10 seconds after a jittered fixation cross (mean 3sec +/- 1sec) (see Fig.1-B). The participant had a four-button response box in their right hand and were instructed to press the "solution" button (right index finger) as soon as they found a solution even though the Mooney image would stay on the screen for the remaining time. The Mooney images were presented as an invariant 10 second block to be able to compare time series of equal length for subsequent functional connectivity analyses. If the participants did not press the "solution" button during the 10 second stimulus presentation, a new trial would start, and the participants were instructed to press a "no solution" button (middle finger) as soon as they saw the fixation cross to keep the amount of button presses constant for each trial. If the participants did press the "solution" button during stimulus presentation, they would be first presented with a jittered fixation cross (mean 3.5sec +/- 1.5sec), followed by four questions regarding the solution and their insight experience: First, four out of 13 broad categories, e.g. "mammal", "human being", "body & health" or "objects in the house" (see Supplementary Methods) were shown and the participants had to indicate the category of their solution. On average, they needed 3.13sec (SD = 2.5sec) to make a response. This way of assessing the solution was used to test whether participants correctly identified the Mooney object, because they could not type their response in the scanner. Subsequently, they were asked about their suddenness (On a scale from 1 – 4: Did the solution come to you in a sudden or more gradual manner?), emotional response (On a scale from 1 – 4: How strong was your positive emotional response upon solution?") and certainty ("On a scale from 1 – 4: How certain are you that the solution is correct?") regarding the solution on a 4-point Likert scale. There was no time limit for these 4 questions, and subsequently, the new trial would start. Note, the 30 Mooney images per run were presented interleaved together with 30 anagram riddles which are not part of the current study. Note also, the experimental blinding for this study does not apply because the participants themselves generated the respective conditions (HI-I and LO-I) during the experiment depending on how they rated their Aha experience.

Five days after the second fMRI session (4.62 days [SD=0.49]), participants completed the subsequent memory test online at home using the Inquisit Software (v.4.0; https://www.millisecond.com/). They were instructed to complete the task independently and were not informed beforehand that they would later need to perform a memory task. All 120 Mooney images they had seen in the scanner were randomly presented mixed with the 60 new Mooney images they had not seen before. Participants were explicitly instructed not to identify the Mooney objects again but only rely on their memory. In each trial the respective Mooney image was shown after fixation cross (one sec) for maximally eight seconds. During this time, participants had to decide whether they had seen this Mooney image before during the MRI sessions, i.e. whether they recognized the high-contrast black and white picture. In particular, they were asked to rate their confidence on a 5-point scale (definitely old, probably old, don't know, probably new, definitely new). If they responded 'don't know', 'probably new' or 'definitely new', the next trial would start. If they responded old (definitely old, probably old), they were asked whether they had identified this Mooney object before in the scanner again using a 5-point confidence scale without a time limit (definitely solved, probably solved, don't know, probably not solved, definitely not solved). If they responded 'solved' (definitely solved or probably solved), they were finally asked to enter the name of the Mooney object (no time limit). Subsequently the next trial would start.

Timing

All fMRI and behavioral data was acquired between April and August 2021.

Data exclusions

We excluded 7 participants from data analysis due to technical issues at the scanner (N=1), excessive head movement in the scanner (N=3), pathological findings in brain anatomy (N=2) and too low performance (0% correct recall) in the subsequent memory test (N=1) resulting in a final sample of N=31.

Non-participation

No participants dropped out or declined participation once they were invited to the fMRI sessions, that is once they were declared MRI compatible and met all inclusion/exclusion criteria. 8 participants were not invited due to MRI non-compatibility.

Randomization

This was a within-subject design, hence not subjects but trials were randomized and runs were counterbalanced across subjects.

Ecological, evolutionary & environmental sciences study design

All studies must disclose on these points even when the disclosure is negative.

Study description	*Briefly describe the study. For quantitative data include treatment factors and interactions, design structure (e.g. factorial, nested, hierarchical), nature and number of experimental units and replicates.*
Research sample	*Describe the research sample (e.g. a group of tagged Passer domesticus, all Stenocereus thurberi within Organ Pipe Cactus National Monument), and provide a rationale for the sample choice. When relevant, describe the organism taxa, source, sex, age range and any manipulations. State what population the sample is meant to represent when applicable. For studies involving existing datasets, describe the data and its source.*
Sampling strategy	*Note the sampling procedure. Describe the statistical methods that were used to predetermine sample size OR if no sample-size calculation was performed, describe how sample sizes were chosen and provide a rationale for why these sample sizes are sufficient.*
Data collection	*Describe the data collection procedure, including who recorded the data and how.*
Timing and spatial scale	*Indicate the start and stop dates of data collection, noting the frequency and periodicity of sampling and providing a rationale for these choices. If there is a gap between collection periods, state the dates for each sample cohort. Specify the spatial scale from which the data are taken*
Data exclusions	*If no data were excluded from the analyses, state so OR if data were excluded, describe the exclusions and the rationale behind them, indicating whether exclusion criteria were pre-established.*
Reproducibility	*Describe the measures taken to verify the reproducibility of experimental findings. For each experiment, note whether any attempts to repeat the experiment failed OR state that all attempts to repeat the experiment were successful.*
Randomization	*Describe how samples/organisms/participants were allocated into groups. If allocation was not random, describe how covariates were controlled. If this is not relevant to your study, explain why.*
Blinding	*Describe the extent of blinding used during data acquisition and analysis. If blinding was not possible, describe why OR explain why blinding was not relevant to your study.*

Did the study involve field work? ☐ Yes ☐ No

Field work, collection and transport

Field conditions	*Describe the study conditions for field work, providing relevant parameters (e.g. temperature, rainfall).*
Location	*State the location of the sampling or experiment, providing relevant parameters (e.g. latitude and longitude, elevation, water depth).*
Access & import/export	*Describe the efforts you have made to access habitats and to collect and import/export your samples in a responsible manner and in compliance with local, national and international laws, noting any permits that were obtained (give the name of the issuing authority, the date of issue, and any identifying information).*
Disturbance	*Describe any disturbance caused by the study and how it was minimized.*

Reporting for specific materials, systems and methods

We require information from authors about some types of materials, experimental systems and methods used in many studies. Here, indicate whether each material, system or method listed is relevant to your study. If you are not sure if a list item applies to your research, read the appropriate section before selecting a response.

Materials & experimental systems

n/a	Involved in the study	
☒	☐	Antibodies
☒	☐	Eukaryotic cell lines
☒	☐	Palaeontology and archaeology
☒	☐	Animals and other organisms
☒	☐	Clinical data
☒	☐	Dual use research of concern
☒	☐	Plants

Methods

n/a	Involved in the study	
☒	☐	ChIP-seq
☒	☐	Flow cytometry
☐	☒	MRI-based neuroimaging

Antibodies

Antibodies used	*Describe all antibodies used in the study; as applicable, provide supplier name, catalog number, clone name, and lot number.*
Validation	*Describe the validation of each primary antibody for the species and application, noting any validation statements on the manufacturer's website, relevant citations, antibody profiles in online databases, or data provided in the manuscript.*

Eukaryotic cell lines

Policy information about cell lines and Sex and Gender in Research

Cell line source(s)	*State the source of each cell line used and the sex of all primary cell lines and cells derived from human participants or vertebrate models.*
Authentication	*Describe the authentication procedures for each cell line used OR declare that none of the cell lines used were authenticated.*
Mycoplasma contamination	*Confirm that all cell lines tested negative for mycoplasma contamination OR describe the results of the testing for mycoplasma contamination OR declare that the cell lines were not tested for mycoplasma contamination.*
Commonly misidentified lines (See ICLAC register)	*Name any commonly misidentified cell lines used in the study and provide a rationale for their use.*

Palaeontology and Archaeology

Specimen provenance	*Provide provenance information for specimens and describe permits that were obtained for the work (including the name of the issuing authority, the date of issue, and any identifying information). Permits should encompass collection and, where applicable, export.*
Specimen deposition	*Indicate where the specimens have been deposited to permit free access by other researchers.*
Dating methods	*If new dates are provided, describe how they were obtained (e.g. collection, storage, sample pretreatment and measurement), where they were obtained (i.e. lab name), the calibration program and the protocol for quality assurance OR state that no new dates are provided.*

☐ Tick this box to confirm that the raw and calibrated dates are available in the paper or in Supplementary Information.

Ethics oversight	*Identify the organization(s) that approved or provided guidance on the study protocol, OR state that no ethical approval or guidance was required and explain why not.*

Note that full information on the approval of the study protocol must also be provided in the manuscript.

Animals and other research organisms

Policy information about studies involving animals; ARRIVE guidelines recommended for reporting animal research, and Sex and Gender in Research

Laboratory animals	*For laboratory animals, report species, strain and age OR state that the study did not involve laboratory animals.*
Wild animals	*Provide details on animals observed in or captured in the field; report species and age where possible. Describe how animals were caught and transported and what happened to captive animals after the study (if killed, explain why and describe method; if released, say where and when) OR state that the study did not involve wild animals.*
Reporting on sex	*Indicate if findings apply to only one sex; describe whether sex was considered in study design, methods used for assigning sex. Provide data disaggregated for sex where this information has been collected in the source data as appropriate; provide overall numbers in this Reporting Summary. Please state if this information has not been collected. Report sex-based analyses where performed, justify reasons for lack of sex-based analysis.*
Field-collected samples	*For laboratory work with field-collected samples, describe all relevant parameters such as housing, maintenance, temperature, photoperiod and end-of-experiment protocol OR state that the study did not involve samples collected from the field.*
Ethics oversight	*Identify the organization(s) that approved or provided guidance on the study protocol, OR state that no ethical approval or guidance was required and explain why not.*

Note that full information on the approval of the study protocol must also be provided in the manuscript.

Clinical data

Policy information about clinical studies

All manuscripts should comply with the ICMJE guidelines for publication of clinical research and a completed CONSORT checklist must be included with all submissions.

Clinical trial registration	*Provide the trial registration number from ClinicalTrials.gov or an equivalent agency.*
Study protocol	*Note where the full trial protocol can be accessed OR if not available, explain why.*
Data collection	*Describe the settings and locales of data collection, noting the time periods of recruitment and data collection.*
Outcomes	*Describe how you pre-defined primary and secondary outcome measures and how you assessed these measures.*

Dual use research of concern

Policy information about dual use research of concern

Hazards

Could the accidental, deliberate or reckless misuse of agents or technologies generated in the work, or the application of information presented in the manuscript, pose a threat to:

No	Yes	
☒	☐	Public health
☒	☐	National security
☒	☐	Crops and/or livestock
☒	☐	Ecosystems
☒	☐	Any other significant area

Experiments of concern

Does the work involve any of these experiments of concern:

No	Yes	
☒	☐	Demonstrate how to render a vaccine ineffective
☒	☐	Confer resistance to therapeutically useful antibiotics or antiviral agents
☒	☐	Enhance the virulence of a pathogen or render a nonpathogen virulent
☒	☐	Increase transmissibility of a pathogen
☒	☐	Alter the host range of a pathogen
☒	☐	Enable evasion of diagnostic/detection modalities
☒	☐	Enable the weaponization of a biological agent or toxin
☒	☐	Any other potentially harmful combination of experiments and agents

Plants

Seed stocks	*Report on the source of all seed stocks or other plant material used. If applicable, state the seed stock centre and catalogue number. If plant specimens were collected from the field, describe the collection location, date and sampling procedures.*
Novel plant genotypes	*Describe the methods by which all novel plant genotypes were produced. This includes those generated by transgenic approaches, gene editing, chemical/radiation-based mutagenesis and hybridization. For transgenic lines, describe the transformation method, the number of independent lines analyzed and the generation upon which experiments were performed. For gene-edited lines, describe the editor used, the endogenous sequence targeted for editing, the targeting guide RNA sequence (if applicable) and how the editor was applied.*
Authentication	*Describe any authentication procedures for each seed stock used or novel genotype generated. Describe any experiments used to assess the effect of a mutation and, where applicable, how potential secondary effects (e.g. second site T-DNA insertions, mosiacism, off-target gene editing) were examined.*

ChIP-seq

Data deposition

- [ ] Confirm that both raw and final processed data have been deposited in a public database such as GEO.
- [ ] Confirm that you have deposited or provided access to graph files (e.g. BED files) for the called peaks.

Data access links<br>*May remain private before publication.*	*For "Initial submission" or "Revised version" documents, provide reviewer access links. For your "Final submission" document, provide a link to the deposited data.*
Files in database submission	*Provide a list of all files available in the database submission.*
Genome browser session<br>(e.g. UCSC)	*Provide a link to an anonymized genome browser session for "Initial submission" and "Revised version" documents only, to enable peer review. Write "no longer applicable" for "Final submission" documents.*

Methodology

Replicates	*Describe the experimental replicates, specifying number, type and replicate agreement.*
Sequencing depth	*Describe the sequencing depth for each experiment, providing the total number of reads, uniquely mapped reads, length of reads and whether they were paired- or single-end.*
Antibodies	*Describe the antibodies used for the ChIP-seq experiments; as applicable, provide supplier name, catalog number, clone name, and lot number.*
Peak calling parameters	*Specify the command line program and parameters used for read mapping and peak calling, including the ChIP, control and index files used.*
Data quality	*Describe the methods used to ensure data quality in full detail, including how many peaks are at FDR 5% and above 5-fold enrichment.*
Software	*Describe the software used to collect and analyze the ChIP-seq data. For custom code that has been deposited into a community repository, provide accession details.*

Flow Cytometry

Plots

Confirm that:

- [ ] The axis labels state the marker and fluorochrome used (e.g. CD4-FITC).
- [ ] The axis scales are clearly visible. Include numbers along axes only for bottom left plot of group (a 'group' is an analysis of identical markers).
- [ ] All plots are contour plots with outliers or pseudocolor plots.
- [ ] A numerical value for number of cells or percentage (with statistics) is provided.

Methodology

Sample preparation	*Describe the sample preparation, detailing the biological source of the cells and any tissue processing steps used.*
Instrument	*Identify the instrument used for data collection, specifying make and model number.*
Software	*Describe the software used to collect and analyze the flow cytometry data. For custom code that has been deposited into a community repository, provide accession details.*
Cell population abundance	*Describe the abundance of the relevant cell populations within post-sort fractions, providing details on the purity of the samples and how it was determined.*
Gating strategy	*Describe the gating strategy used for all relevant experiments, specifying the preliminary FSC/SSC gates of the starting cell population, indicating where boundaries between "positive" and "negative" staining cell populations are defined.*

- [ ] Tick this box to confirm that a figure exemplifying the gating strategy is provided in the Supplementary Information.

Magnetic resonance imaging

Experimental design

Design type	task; event-related and block-design

Design specifications	Participants had two MRI sessions on two consecutive days and in each session they completed two runs with 30 trials each. Each run took ca. 15-25min. Between the two runs (in each session) there was 10-15min break.
Behavioral performance measures	We recorded the solution button press, response time for this button press, as well as the insight ratings and a categorization task (to estimate accuracy for the solution) (see Method section).<br>Insight was estimated by summing up the individual insight ratings and using a median split dividing the trials in high or low insight trials.<br>Estimated marginal means and standard deviations were used to calculate differences between accuracy and response times between high and low insight trials.

Acquisition

Imaging type(s)	functional and structural
Field strength	3
Sequence & imaging parameters	Task-based functional (henceforth fMRI) and structural images were collected on a Siemens Magnetom Prisma 3T scanner (Erlangen, Germany) and a standard 64-channel head coil was used. All sequences were adapted according to the Human Connective Project (Harms et al., 2018). Multiband functional images were collected using a T2*-weighted echo planar imaging (EPI) sequence sensitive to blood oxygen level dependent (BOLD) contrast (TR=0.8ms; TE=37ms, voxel size=2.0mm³, flip angle=52°, FoV=208mm, acquisition matrix = 208×208×144; 72 slices).<br><br>The structural images were obtained using a three-dimensional T1-weighted magnetization prepared gradient-echo sequence (MPRAGE) (TR = 2500ms; TE = 2.22ms; TI = 1000ms, acquisition matrix =240×256×167, FoV=256mm, flip angle = 8°; 0.8 mm³ voxel size, 208 slices). Additionally a spin echo field map was acquired to account for the B0 inhomogeneities (TR=8000ms; TE=66ms; flip angle =90°, 72 slices; FoV=208mm; 2mm³ voxel size, acquisition matrix = 208x208x144).
Area of acquisition	whole brain

Diffusion MRI ☐ Used ☒ Not used

Preprocessing

Preprocessing software	Preprocessing was done via fMRIPrep version 20.2.5 using default parameters (without slice time correction due to short TR= 800ms)
Normalization	Spatial normalisation to the ICBM 152 Nonlinear Asymmetrical template version 2009c [Fonov et al., 2009; RRID:SCR_008796] was performed through nonlinear registration with the antsRegistration tool of ANTs v2.1.0 [(Avants et al., 2008), RRID:SCR_004757], using brain-extracted versions of both T1w volume and template.
Normalization template	ICBM 152 Nonlinear Asymmetrical template version 2009c<br>Note, for control analysis, univariate data analysis of amygdala and hippocampus was additionally performed in native subject T1w space (see Supplement).
Noise and artifact removal	Noise and artifact removal was done via fMRIPrep (default parameters:)<br>Functional data was motion corrected using MCFLIRT (FSL v5.0.9, (Jenkinson et al., 2002). Distortion correction was performed using an implementation of the TOPUP technique (Andersson et al., 2003) using 3dQwarp [AFNI v16.2.07; (Cox, 1996)]. This was followed by co-registration to the corresponding T1w using boundary-based registration (D. N. Greve & Fischl, 2009) with six degrees of freedom, using bbregister (FreeSurfer v6.0.1). Motion correcting transformations, field distortion correcting warp, BOLD-to-T1w transformation and T1w-to-template (MNI) warp were concatenated and applied in a single step using antsApplyTransforms (ANTs v2.1.0) using Lanczos interpolation.<br>Physiological noise regressors were extracted by applying anatomical CompCor (aCompCor) (Behzadi et al., 2007). Here, six components were calculated within the intersection of the subcortical mask and the union of CSF and WM masks calculated in T1w space, after their projection to the native space of each functional run. Framewise displacement (Power et al., 2014) was calculated for each functional run using the implementation of Nipype.
Volume censoring	-

Statistical modeling & inference

Model type and settings	We used univariate, RSA and functional connectivity fMRI analyses including graph measures to test our hypotheses. The methods for every analysis type (univariate, RSA etc.), their stimulus conditions and the respective statistical tests used (usually generalized linear mixed models) are described in detail in the Method section.
Effect(s) tested	Insight Measure. Recent studies showed that the overall subjective insight (AHA) experience is continuous and can be decomposed into three different dimensions: (1) positive emotional response or internal reward upon finding the solution, (2) experienced suddenness of the solution and (3) certainty about the solution (Danek & Wiley, 2017, 2020). To have better control over these three dimensions, we assessed them separately, and then combined them into a compound insight measure (see section: Mooney image paradigm) using a continuous approach as well as binary one (for functional connectivity/graph analyses and behavioral data for demonstration purposes). In the "insight median split" (binary) approach, we added up the ratings of positive emotion, suddenness, and certainty (1-4 each, 3-12 for the total), and used a median split of this measure to distinguish high- and low-insight trials. The advantage of this approach is that it yields a

roughly equal number of trials for high- and low-insight, but its disadvantage is it is not the most sensitive insight measure as it is binarized. Thus, in the "insight sum" (continuous) approach, we added up the ratings of positive emotion, suddenness and certainty and used this as a continuous measure for insight. Both measures produced similar results except for where it is specifically stated in the results section.

Subsequent Memory Measure. We were more interested in recall (naming the correct Mooney object) than just recognition (remembering to have seen the black/white Mooney dots) because in the context of problem solving the more relevant memory measure is the ability to recall the content of the solution than just to recognize the problem, i.e. Mooney object. To reduce the likelihood that participants just identify the Mooney image again instead of remembering the depicted object, we used all three questions as a compound memory measure. Therefore, the identification of the Mooney object was considered as remembered, when it was identified in the MR scanner and 1) the Mooney image was correctly recognized as seen in the scanner, 2) the Mooney image was correctly recognized as having been identified in the scanner and finally 3) the hidden Mooney object was correctly named in the Subsequent Memory Test (see section Subsequent Memory Test).

We used generalized linear mixed models to investigate the effect of insight on subsequent memory and univariate BOLD activity as well as RSA, functional connectivity and graph measures (see Method section). Due to the relatively lower trial count for the insight-memory analyses we used a non-parametric bootstrapping approach with 1,000 resamples to estimate the odds ratios and their 95% confidence intervals for the effects of interest. To enhance statistical robustness for the fMRI-related insight-memory analyses (except for connectivity/graph analyses), we implemented permutation tests to derive p-values from comparing nested random effects models (permlmer) using the predictmeans package (v.1.1.0) in R, with 999 permutations (Lee & Braun, 2012).

Specify type of analysis: ☐ Whole brain ☐ ROI-based ☒ Both

Anatomical location(s)

We used the marsbar toolbox with its default parameter settings to extract the averaged beta values representing the respective onset regressors from the ROIs per session and block (Brett et al., 2002). The left and right ROI masks for amygdala and hippocampus were extracted from the FSL Harvard-Oxford (HO) Atlas (automated labeling) (Desikan et al., 2006). Furthermore due to functional differences, the hippocampus mask was divided into an anterior and posterior part based on the conventional landmark of the uncal apex (y = -21/22 in MNI space, Poppenk et al., 2013). To check that normalization of each individual worked well and the ROI masks overlap with the respective amygdala and hippocampus position in the brain, we additionally visually inspected every T1 image with the respective ROI masks.

To exclude potential normalisation artefacts due to the small volume of the amygdala and hippocampal ROIs, we additionally conducted another ROI analysis in subject space for the contrast HI-I>LO-I. Functional and structural data were preprocessed using fMRIprep in the same way as for the analysis in standard space (see Methods section fMRI data preprocessing), with the exception that data were resampled into the individual's anatomical reference generated with the T1 weighted images. All further analyses (first and second level analyses, beta value extraction via marsbar) were identical to the analyses reported for the data in standard space further above. The only exception was that the amygdala and the anterior and posterior hippocampus ROIs were first resampled into subject-space using the ANTs function antsApplyTransforms (Avants et al., 2009) before extracting the condition specific mean beta values using marsbar. Visual inspection was used to test that the resampled ROIs masks matched the position of respective brain regions in subject space for each individual.

We also provide a whole-brain analyses in the supplement for exploratory purposes to examine activity that is parametrically modulated by the intensity of the Aha! experience. For this we modelled the event of the solution button press (onset regressor; the rest of the analysis is identical to the ROI analysis).

Statistic type for inference

(See Eklund et al. 2016)

For the whole-brain analyses, we used SPM's one-sample t-test with the following multiple comparison correction: voxel-level threshold of p<.001 and a cluster-level threshold of p<.05 (family-wise error corrected).

Correction

Where it applies, we, FWE, FDR or Bonferroni corrected to adjust for multiple comparison between the ROIs.

Models & analysis

n/a	Involved in the study
☐	☒ Functional and/or effective connectivity
☐	☒ Graph analysis
☐	☒ Multivariate modeling or predictive analysis

Functional and/or effective connectivity

We expected insight-related increased functional connectivity between those areas that process the solution object (VOTC) and those that likely evaluate it such as the amygdala and hippocampus. To estimate functional connectivity (FC) between those areas, we used the fMRIprep preprocessed fMRI data (see above) and first denoised and subsequently analysed it in CONN - an open source connectivity toolbox (version 21.a)111.
Denoising. To remove confounding effects to the estimated BOLD signal for each subject in each voxel and at each time point, i.e. session and block, CONN's implemented anatomical component-based noise correction procedure (CompCor) was used. Those confounding effects involve six subject-motion parameters including their first-order derivatives, identified outlier scans, constant or linear condition effects as well as noise components from areas of cerebral white matter and cerebrospinal fluid. Note, to avoid the risk of artificially introducing anticorrelations (negative connectivity) into the FC estimates, no global signal regression was

applied112. Finally, the resulting time series were band-pass filtered to .008 - .09 Hz.
First-level analysis. FC during high versus low insight conditions (HI-I>LO-I) was estimated. We were only interested in ROIs processing the solution object and therefore constructed a specific solution network comprising left and right aFusG, pFusG, aITG, mITG, pITG, iLOC including left and right amygdala and anterior as well as posterior hippocampus (18 ROIs in total). Amygdala and hippocampus were included because we were additionally interested in whether those brain areas that showed insight-related differences in univariate BOLD activity are also part of this solution network. All ROIs taken from the FSL Harvard Oxford Atlas were identical to the ones already used for univariate and the other multivariate analyses but analysed separately by hemisphere.53,54 The time series for each ROI were acquired by averaging the BOLD time series of each voxel belonging to the respective ROI. ROI-to-ROI connectivity matrices (solution network: 18x18) were computed for each participant (31), for each condition (1) HI-I vs. LO-I, each session (2) and each block (2) separately. Each element in each connectivity matrix represents the strength of FC between a pair of ROIs and was defined as the Fisher-transformed bivariate correlation coefficient between the preprocessed and denoised BOLD time series of two ROIs.
Second-level analysis. To estimate FC for the contrast HI-I>LO-I in the solution network, the within-subjects contrast in the GLM was specified as follows y~ 1*HI-I – 1*LO-I + ε). For statistical inference, the 18x18 ROI connectivity matrix was thresholded at the connection-level at p<.05 (p-uncorrected, one-sided [we only expected positive connectivity]) and at the network-level at p<.05 (ROI-p, FWE corrected). Note, for cluster-level inference we adopted the Network Based Statistics (NBS) approach113.
To further explore whether FC in the solution network also predicts insight-related better memory, we estimated another first level analysis identical to the one described above with the only difference that four different conditions were estimated: 1) high insight - remembered trials [HI-I-Rem], 2) high insight - forgotten trials [HI-I-Forg], 3) low insight - remembered trials [LO-I-Rem] and 4) low insight - forgotten trials [LO-I-Forg]. For the second level analysis, we specified the following within-subjects contrast in the GLM: y~ 1*HI-I-Rem - 1*HI-I-Forg - 1*LO-I-Rem + 1*LO-I-Forg + ε.

Graph analysis

To further characterise efficient information integration during insight we computed several network measures quantifying functional integration (increased global efficiency, reduced average path length), segregation (increased local efficiency and clustering coefficient) and centrality (degree) of the solution network and its individual ROIs with the contrast HI-I>LO-I as implemented in CONN111. Note, the description and mathematical formulas for each graph measure is in Table S2.
All ROI-graph measures were based on functional connectivity values of our pre-defined non-directed solution network (18x18 Fisher transformed correlation matrix). For each subject and condition (HI-I, LO-I) a series of adjacency matrices are then computed by thresholding the respective Fisher transformed correlation matrix by different correlation coefficients ranging from r=0.10 to r=0.8 in steps of 0.1. Note, only positive correlations were included for better interpretation of the subsequent graph measures52. From those resulting adjacency matrices, several graph-theoretical network measures of interest were subsequently computed for the contrast HI-I>LO-I addressing topological properties of each ROI within the matrix but also of the entire solution network. Importantly, to demonstrate the robustness of the results rather than just picking an arbitrary threshold, we aggregated across the different adjacency matrices (acquired via those different correlation coefficient thresholds) to compute the respective graph measure of interest. Note, we tested one-sided due to directed hypotheses.

Multivariate modeling and predictive analysis

Representational similarity analyses (this section is copy-pasted from the Method section)

We hypothesised that 1) RC should be associated with changes in distributed, i.e. multivoxel, patterns from pre- to post-solution, 2) those patterns should start representing the meaningful solution object after solution and 3) visual RC in Mooney images (perceptual regrouping of black/white contours leading to sudden object recognition) should occur in regions involved in visual object recognition along the visual ventral pathway such as the temporal-occipital [pITG], anterior [aITG] and posterior [mITG] Inferior Temporal Gyrus; inferior Lateral Occipital Cortex [iLOC]; anterior, posterior, occipito-temporal [toFusG] and occipital [oFusG] Fusiform Gyrus42,93,94. The masks for those ROIs were extracted from the FSL Harvard-Oxford (HO) Atlas95. Note, to reduce the number of Fusiform Gyrus ROIs but respect functional differences96, we merged this region into only two ROIs: anterior [aFusG] and posterior [pFusG] Fusiform Gyrus. To create the aFusG, the anterior and posterior Fusiform Gyrus ROIs from the HO Atlas were merged into one. To create the pFusG, the toFusG and oFusG ROIs were merged into one.
We used RSA to test whether and which ROIs along the visual ventral pathway (VOTC areas) exhibit those properties during insightful problem solution. We first extracted the multivoxel pattern in the above named ROIs per subject, per Mooney image and for a pre- as well as post-solution time point. Subsequently, we conducted two different types of representational similarity analysis: 1) pre- to post-solution similarity to identify ROIs showing a stronger decrease in correlation for HI-I than LO-I trials and 2) a model-based RSA20,37 where distributed activity patterns were correlated with the output of two very distinct neural network models, AlexNet and Word2Vec.
Extraction of ROI-wise multivoxel patterns. To obtain multivoxel patterns, we first estimated beta values in each voxel for each pre- and post-solution event. Due to the short response time in particular for HI-I events, the pre- and post-solution regressors would be highly collinear, i.e. explain shared variance of the time-series, which results in unreliable parameter estimation and an hence an underestimation of the similarity of activity patterns97. Therefore, we conducted two separate first-level models - one for the pre- and one for the post-solution time points in which each event was represented by an individual regressor (its onset convolved with the HRF). Pre-solution onsets were 0.5 sec after onset of the Mooney images and post-solution onsets were the time point of the button presses; both events were modelled with a duration of 1 sec. Because there was no button press for unsolved trials during the stimulus presentation, we created a jittered onset regressor for the “post-solution stage” of the unsolved trials that on average matched the response time of the solved trials (~3.7sec). These two univariate single-trial models were conducted as general linear models as implemented in SPM. Due to the sluggish HRF, one would expect a higher intrinsic auto-correlation of the activity patterns of pre and post events when they occur close in time which is on

average more often the case for HI-I than LO-I events. Importantly though, this auto-correlation rather counteracts the hypothesised greater decrease in similarity in HI-I trials because they were solved faster and therefore do not confound the results. We added an additional nuisance regressor for all remaining button presses (related to the insight ratings and selecting a solution category) for one second starting with the respective button press. Additionally, we separately modelled six motion parameters and separately modelled the mean for each of the four runs. The time series were corrected for baseline drifts by applying a high-pass filter (128 sec) and for serial dependency by an AR(1) autocorrelation model.
Subsequently, the corresponding pre- and post-solution activation patterns for each Mooney image from the corresponding beta images per ROI (iLOC, aFusG, pFusG, aITG mITG, pITG) were extracted and vectorized. Those ROI-wise multivoxel patterns were used for 1) Pre- to post-solution RSA and 2) Pre- to post-solution model-based RSA. All analyses were conducted using in-house MATLAB scripts.
1. Pre- to post-solution RSA. To compare the trial-wise change in multivoxel pattern from pre- to post-solution, we first matched the extracted pre- and post-solution activation patterns for each Mooney image from the corresponding beta images per ROI into a pre- to post solution vector pair and subsequently correlated it (Spearman's Rho) (see Fig.3-A).
For statistical analysis, a linear mixed effects model was applied estimating the multivoxel pattern similarity between the pre- and post-solution phase (dependent variable) as a function of the insight (continuous) for correctly solved trials and respective ROI. Additionally, response time and the run order (1-4) were added as covariate of no interest, and we included only trials where pre- and post-solution phases were at least 2 seconds apart to reduce overlap of the hemodynamic response. Item and subject variables were estimated as random intercepts. To estimate differences in multivoxel pattern similarity between ROIs as a function of insight, another model was estimated including an interaction term between ROI and insight (see equations 3-5 below).
(3) PPSS ~ RT+ run + ROI + (1| ID) + (1|item) + Ɛ
(4) PPSS ~ RT+ run + ROI + insight + (1| ID) + (1|item) + Ɛ
(5) PPSS ~ RT+ run + ROI * insight + (1| ID) + (1|item) + Ɛ
Note. PPSS = Pre-Post Solution Similarity; RT = response time.

For the resulting p-values, we used Bonferroni-corrected alpha levels because we did not have a hypothesis which ROI may exhibit insight-related changes in multivoxel pattern similarity.

2. Pre- to post-solution model-based RSA. Here, representational similarity between the activity pattern elicited by the stimuli is compared to the computationally model-based representational similarity between the same stimuli. This analysis involves four steps: 1) creating representational similarity matrices for each ROI based on the correlation of the multivoxel activity patterns elicited by the stimuli, 2) creating a model-based similarity matrix based on the correlation of the output of the computational model for each stimulus 3) correlating the model-based similarity matrix with the ROI-specific representational similarity matrices for each time point 4) associating the brain-model correlation for each trial with insight or insight*memory.

1) Creating Neural Activity-derived RSMs (Brain RSM)
For each subject, ROI (iLOC, aFusG, pFusG, aITG, mITG, pITG) and time point (pre- and post solution), a Neural activity-derived RSM (120x120) was created representing the similarity in the multivoxel activity pattern across stimuli. That is to say, each cell in a matrix represents the Spearman correlation between the vectorized multivoxel patterns of two Mooney images (see Fig.3-B1).

2) Creating Model RSMs
Because we assumed that conceptual similarities between the Mooney objects increases after problem solution (=object identification), we created a Model RSM consisting of pair-wise conceptual similarity values for each Mooney image pair represented in a 120x120 matrix. To assure robustness of the results, we used two different methods to quantify conceptual similarity between the Mooney images - a deep convolutional network (DNN) AlexNet49,98,99 and a Word2Vec model45,48,100 resulting in two different conceptual Model RSMs.
Conceptual similarity via the DNN AlexNet Model RSM is based on categorical features49. DNNs consist of layers of convolutional filters that are trained to classify images into categories with a high level of accuracy. While filters from early layers predominately detect simple visual features, later layers organise items by their categorical features101. According to previous research, DNNs like AlexNet model visual representations along the ventral visual pathway98,99 and outperform traditional theoretical models of the ventral visual pathway (e.g., HMAX, object-based models)102,103 in their capacity to identify specific objects with the appropriate category-level label. They may therefore be particularly well suited to investigate RC of visual objects using Mooney images. Here, we used the pre-trained 8-layer DNN, the AlexNet, which was successfully trained to classify 1.2 million images into 1000 categories49. AlexNet consists of eight layers, the first five are convolutional (some are followed by max-pooling layers) and the last three are fully connected layers. Conceptual similarity between the Mooney objects was quantified as follows: First, the actual non-abstracted, colored object images (not their abstracted Mooney counterpart) were entered into the pre-trained AlexNet and the activations of its penultimate layer for each image were extracted and converted into one activation vector (note, activation from this layer can be regarded as conceptual because it is the last layer before the images are explicitly categorised into the trained categories)103,104. Subsequently, for each pair of images, the similarity (Spearman correlation) was computed between the activation vectors resulting in a 120x120 matrix (=Model RSM). For a schematic visualisation of this process, see Fig.3-B2).
In contrast, in the Word2Vec model, pair-wise stimuli similarity is based on cosine similarity derived from statistical co-occurrences in text data via a pre-existing word embedding model as previously published (45,105 for more details). Word embeddings represent words as dense numerical vectors derived from a neural network being trained with a huge text corpus using a word2vec algorithm (see48. Cosine similarity is defined as the angle between two 300 dimensional vectors that represent both stimuli (concepts, e.g. "tiger" and "dog") in this vector space. The Model RSM that was created with Word2Vec consisted of pair-wise conceptual similarity values for each Mooney concept (word) represented in a 120x120 matrix.

3) Computing an item-wise representational strength
Subsequently, the item-wise rows (one item = one Mooney image object) from the Neural-activity-derived RSM for each subject, each time point and each ROI were partially correlated (Pearson's r) with the same item-wise rows of each Model RSM (AlexNet and Word2Vec). Note, to control for the confound of having presented the Mooney images in different scans, i.e. blocks and sessions, those item-wise row correlations between the brain and model data were partialled out by a variable indicating the respective run order (1-4). Such an item-wise approach differs from the classical method of assessing such second-order correlations between brain and model activation patterns106, which typically relate the entire item × item matrix at once (note, this item-wise approach has been successfully applied elsewhere,37. Furthermore, the item-wise row correlations were further filtered into solved and unsolved Mooney images because we assumed that only solved Mooney images (successful object identification) should exhibit conceptual representational similarity among each other. The resulting representational strength ("2nd order correlation") identifies brain regions that process and/or store conceptual representations emphasizing either categorical visual features (AlexNet) or similar meaning based on co-occurrences (Word2Vec). Hence, higher representational strength values in a brain region indicate higher conceptual similarity (see Fig.3-B3).

4) Predicting the representational strength values
The statistical analysis was similar to the pre- to post-solution similarity analysis using mixed effects models in a nested way to estimate the effect of changes in representational similarity (i.e. representational strength or brain-model fit) as a function of insight after correct solution, ROI and time (see equations below). Time was a factor that indicated the pre- (0.5sec after stimulus presentation) or the post-solution (solution button press) phase. Note, the ROI factor only contained pFusG and iLOC because only those visual brain areas exhibited changes in pre- to post solution similarity (see 1.). Additionally, response time (RT) and run order were added as covariate of no interest and we included only trials where pre- and post-solution phases were at least 2 seconds apart to minimize overlap of the hemodynamic response. Next to the ROI*insight interaction, we additionally modelled a 3-way interaction (ROI*insight*time) to test which ROI exhibits insight-related changes in representational similarity from pre- to post-solution (IV., see equations below).
(6) Rep. Strength ~ RT+ run + time + ROI + (1|ID) + (1|item) + Ɛ
(7) Rep. Strength ~ RT+ run + time + ROI + insight + (1|ID) + (1|item) + Ɛ
(8) Rep. Strength ~ RT+ run + time + ROI * insight + (1|ID) + (1|item) + Ɛ
(9) Rep. Strength ~ RT+ run + time * ROI * insight + (1|ID) + (1|item) + Ɛ
Note. Rep.Strength = Representational Strength, i.e. brain-model fit.

Finally, we tested whether a) differences in pre to post solution multivoxel pattern similarity as well as b) the representational strength of the solution object after correct solution in those ROIs that show all described properties of RC (pFusG, iLOC) are related to insight-related better memory. To test this, we performed another set of nested model comparisons with a) pre to post solution similarity (PPSS) and b) the changes in representational strength (Rep.Strength: AlexNet or Word2Vec) from pre to post solution as dependent variables and ROI as well as an interaction between (time and) insight and memory as independent factors (see equations below). Note, only pFusG and iLOC exhibited all properties of RC, for this reason we only estimated the described models for those two ROIs. Response time and the run order were entered as covariates of no interest and items and subjects were estimated as random intercepts. To test for differences in insight-related memory between ROIs, another model with ROI*insight*memory interaction term was additionally modelled ((13), see equations below). To enhance statistical robustness for the insight-memory analyses (see Fig. S5), we implemented permutation tests to derive p-values from comparing nested random effects models (permlmer) using the predictmeans package (v.1.1.0) in R, with 999 permutations.55
(10) PPSS ~ RT+ run + ROI + insight + (1| ID) + (1|item) + Ɛ
(11) PPSS ~ RT+ run + ROI + insight + memory + (1| ID) + (1|item) + Ɛ
(12) PPSS ~ RT+ run + ROI + insight * memory + (1| ID) + (1|item) + Ɛ
(13) PPSS ~ RT+ run + ROI * insight * memory + (1| ID) + (1|item) + Ɛ
Note. PPSS = Pre-Post Solution Similarity; RT = solution time.

(14) Rep.Strength ~ RT+ run + ROI + time + insight + (1| ID) + (1|item) + Ɛ
(15) Rep.Strength ~ RT+ run + ROI + time + insight + memory + (1| ID) + (1|item) + Ɛ
(16) Rep.Strength ~ RT+ run + ROI + time + insight * memory + (1| ID) + (1|item) + Ɛ
(17) Rep.Strength ~ RT+ run + ROI + time * insight * memory + (1| ID) + (1|item) + Ɛ
(18) Rep.Strength ~ RT+ run + ROI * time * insight * memory + (1| ID) + (1|item) + Ɛ
Note. Rep. Strength = Representational strength of solution object at post solution (button press); RT = solution time.
